# Detection of Pulpal Blood Flow *In Vivo* with Pulse Oximetry in Dogs

**DOI:** 10.3389/fvets.2016.00036

**Published:** 2016-05-20

**Authors:** Jessica Riehl, Scott J. Hetzel, Christopher J. Snyder, Jason W. Soukup

**Affiliations:** ^1^Dentistry and Oral Surgery Section, Department of Surgical Sciences, School of Veterinary Medicine, University of Wisconsin-Madison, Madison, WI, USA; ^2^Department of Biostatistics and Medical Informatics, University of Wisconsin-Madison, Madison, WI, USA

**Keywords:** dental trauma, diagnosis, pulse oximetry, endodontics, tooth vitality

## Abstract

**Objective:**

The aim of the present study was to investigate the ability of pulse oximetry to detect blood flow within the canine tooth of dogs.

**Materials and methods:**

Dogs aged 18–138 months presenting for oral treatment and meeting the inclusion criteria underwent pulse oximetry evaluation of at least one canine tooth. Oxygen saturation (SpO_2_) and pulse rate values were acquired from vital canine teeth of 38 clinical patients (representing 40 total teeth) with a handheld pulse oximeter and compared to a control area. SpO_2_ values from the tooth and control area were recorded every 5 s for three 60-s intervals. The sensors were repositioned after each 60-s interval.

**Results:**

The vital teeth consistently recorded SpO_2_ values significantly lower than the control area. The mean vital tooth SpO_2_ was 85.9% (median = 87%; SD = 8.6), and the mean control area SpO_2_ was 97.7% (median = 98%; SD = 1.8). An age-related significant difference was noted in vital tooth SpO_2_ values.

**Conclusion:**

In conclusion, the present data support the possible use of pulse oximetry to detect blood flow in canine teeth. However, there was no significant correlation between SpO_2_ values in the vital tooth and in the control areas. Additionally, the results were not definitive, and validation of the modality with additional studies of non-vital canine teeth is needed before clinical use can be recommended.

## Introduction

Diagnostic tests utilized to determine the pulpal vitality in humans conventionally rely upon a series of sensitivity tests that require an immediate qualitative sensory response from the patient and an interpretation of those results by the dentist. Historically, clinical evaluation has involved thermal and electric testing. However, these modalities rely on a neural response and the ability for the patient to acknowledge and express unpleasant sensations. A major shortcoming of conventional sensitivity tests commonly employed in humans is that they are, in actuality, tests of nerve conduction, not pulpal blood flow. Pulp vitality is not dependent upon innervation, but rather on vascular supply. Additionally, those patients with a reduced ability to communicate, such as children, cannot reliably and effectively translate sensations of pain into objective information for the clinician.

Veterinary dentists face an even more profound challenge in the diagnosis of pulpal disease due to the inability of their patients to use verbal language understandable to human intelligence. As a result, veterinarians have historically relied virtually entirely upon radiologic determination of pulpal health. Radiologic features of pulpal necrosis, either a wide root canal diameter (consistent with halted dentinogenesis), tooth root resorption, or the presence of periapical lucency (consistent with periapical periodontitis), are generally considered to be the features of pulpal necrosis that may take months or even years to develop (1). Human studies have shown the sensitivity of radiology alone to determine pulpal necrosis to be moderate at best (2). Consequently, dogs may live with pulpal necrosis and subsequent pain for years before a diagnosis is made.

Discoloration of teeth has been reported to be a predictable indicator of pulpal necrosis (3). The prevalence of pulpal necrosis in discolored canine teeth is purported to be as 92.2% in dogs (3). Whereas in humans, the reported prevalence is much lower at 47–75% (4, 5). Others have noted that tooth discoloration alone does not necessarily indicate pulp necrosis, and veterinary clinicians should be cautious about placing any large degree of certainty on a single study (6–12). However, in most studies, pulpal necrosis has not been substantiated by histopathological confirmation (gold-standard) or through non-invasive quantification of pulpal blood flow. Additionally, as many as 43% of non-discolored, traumatized human primary incisors are necrotic (4). Thus, reliance upon the presence or absence of tooth discoloration as the sole determinant of pulpal vitality may result in a high number of false-negative and false-positive results. To complicate matters further, a common clinical presentation is partial discoloration of the crown. In this scenario, there may be no reliable and objective indication as to whether the pulp is necrotic or vital.

More modern studies in human dentistry have focused on the importance of pulpal vascular circulation by the application of techniques that either directly or indirectly assess the pulpal blood flow, such as laser Doppler flowmetry (LDF) (13–15), dual wavelength spectrophotometry (16), and pulse oximetry (17–26). Although not a direct measurement of blood flow, pulse oximetry is frequently used in veterinary medicine for anesthetic monitoring because it is a non-invasive and simple technique that has the added benefit of providing rapid results. The mechanism of pulse oximetry works on the basic principles of light passing from a photoelectric diode across the tissue to which the device is being applied and reaching a receptor. The differential between light emitted from the diode and light received by the receptor is processed and provides both pulse rate (PR) and oxygen hemoglobin saturation (SpO_2_) values (27).

Several studies have proven that pulse oximetry is effective at assessing pulpal blood flow in humans (20–22, 24–28). Schnettler and Wallace utilized cold and electric stimuli to establish baseline pulpal health in 44 human teeth (22). All teeth, which had positive responses to cold and electric stimuli, also exhibited consistent pulse oximetry values. More recently, Pozzobon et al. confirmed that pulse oximetry could be applied to detect blood flow and vascular integrity in teeth, although specificity was decreased compared to cold and electric stimuli testing (28).

In summary, an objective and accurate clinical method of assessing pulpal vitality is needed in order to reduce false-positive and false-negative results. Pulse oximetry can effectively evaluate and detect pulpal blood flow in humans and may serve as an objective, quantitative diagnostic modality that may compliment, or even replace, current diagnostic methods for determining pulpal vitality in animals. Pulse oximetry, if found effective, would serve as an ideal diagnostic tool due to rapid results, ease of use, non-invasive capabilities, and wide availability. In this study, we aim to evaluate the use of pulse oximetry to detect the blood flow in vital canine teeth. As the ability of pulse oximetry to detect values is largely dependent on the ability of light to penetrate dentin, we hypothesize that patient age may be a limiting factor. Thus, we also aim to study the influence of patient age, as a relative indicator of dentin thickness, on detection ability.

## Materials and Methods

The study involved client owned animals. Clients were informed of the study aims verbally and in writing. Subsequently, clients offered consent and signed a university IACUC approved consent form. The animal care and use protocol utilized for the present study was in accordance with the IACUC standards and was approved by the Animal Care and Use Committee of the University of Wisconsin-Madison School of Veterinary Medicine. Dogs that were presented to the University of Wisconsin-Madison School of Veterinary Medicine Veterinary Medical Teaching Hospital for oral treatment between January 2012 and December 2014 were evaluated for potential study inclusion. Inclusion criteria required patients to be over 11 months of age and possess at least two canine teeth free of caries, restorations, developmental defects, mobility, root resorption, periodontal disease, or endodontic disease. Additionally, dogs enrolled in the study were free of history or gross evidence of prior traumatic dentoalveolar injury. Lack of periodontal disease was confirmed with periodontal probing and intraoral radiography. Pulpal health and vitality of at least two canine teeth were assessed by the lack of radiologic evidence of endodontic disease, as determined with intraoral radiography. Radiologic criteria for evidence of pulpal health included the absence of rarefying or sclerosing pattern of periapical lucency, halted dentinogenesis, and tooth resorption. Additionally, patients with obvious gross pathology involving the jaws were also considered ineligible. Only dogs with an American Society of Anesthesiologists Physical Status Classification (29) of 1 were included in the study. Finally, dogs with endogenously or exogenously stained canine teeth were excluded from the study.

**Figure 1:**
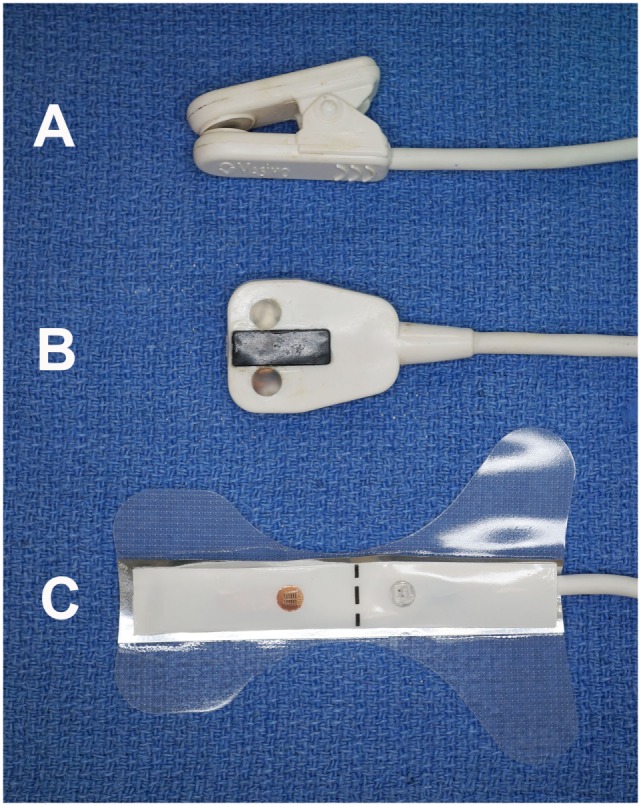
**Photograph of the three sensors utilized in the study**. The clip-tip ear sensor **(A)** was used on the tongue; the adult transflectance forehead sensor **(B)** was used on the gingiva; and the adult adhesive finger sensor **(C)** was used on the teeth.

**Figure 2:**
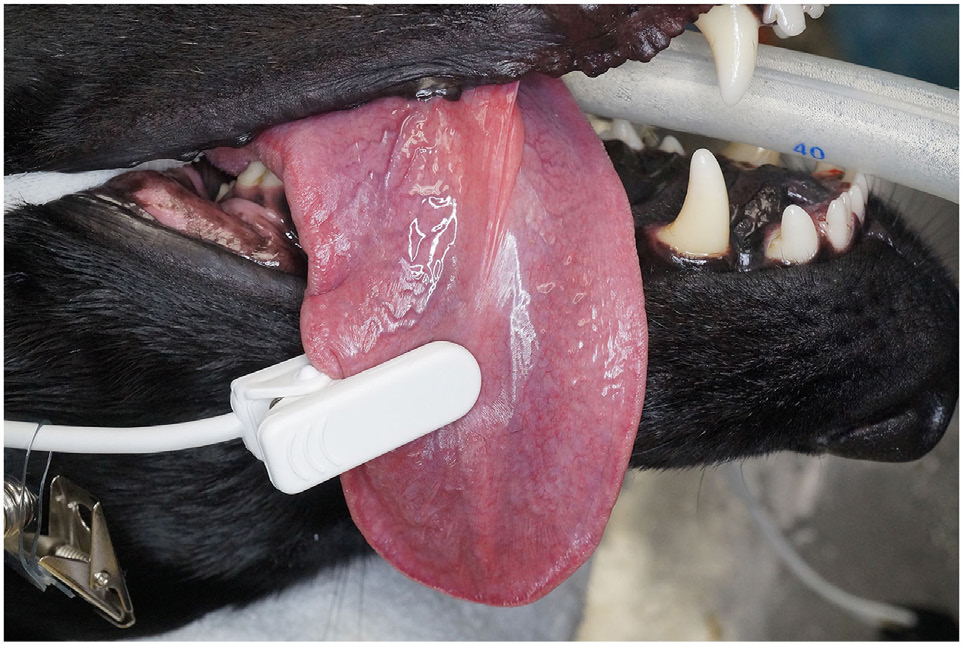
**Photographs depicting the clip-tip sensor placed on the tongue**.

**Figure 3:**
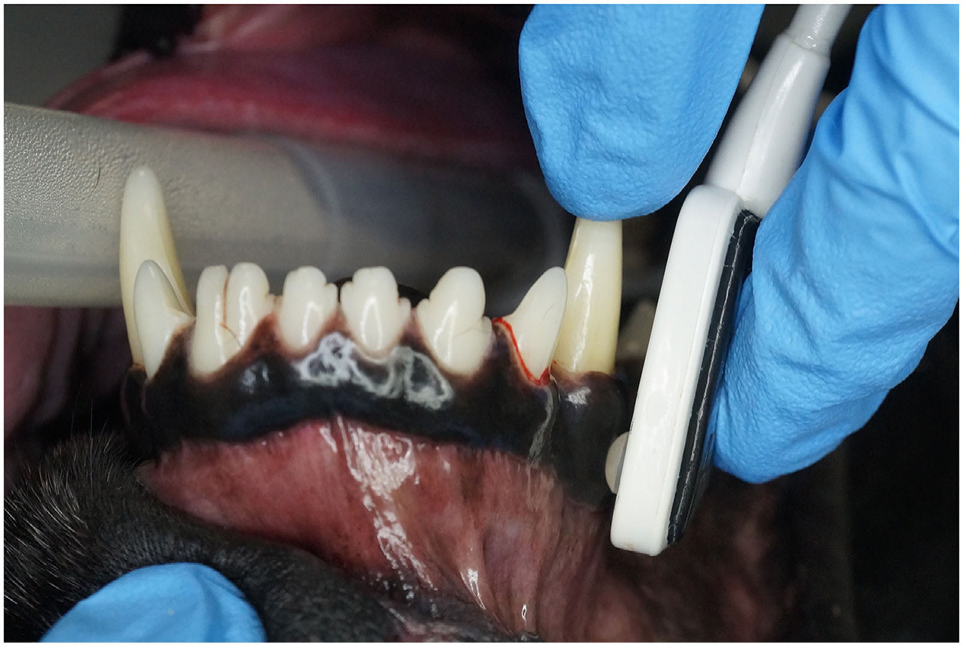
**Photographs depicting the adult transflectance forehead sensor placed on the gingiva**.

**Figure 4:**
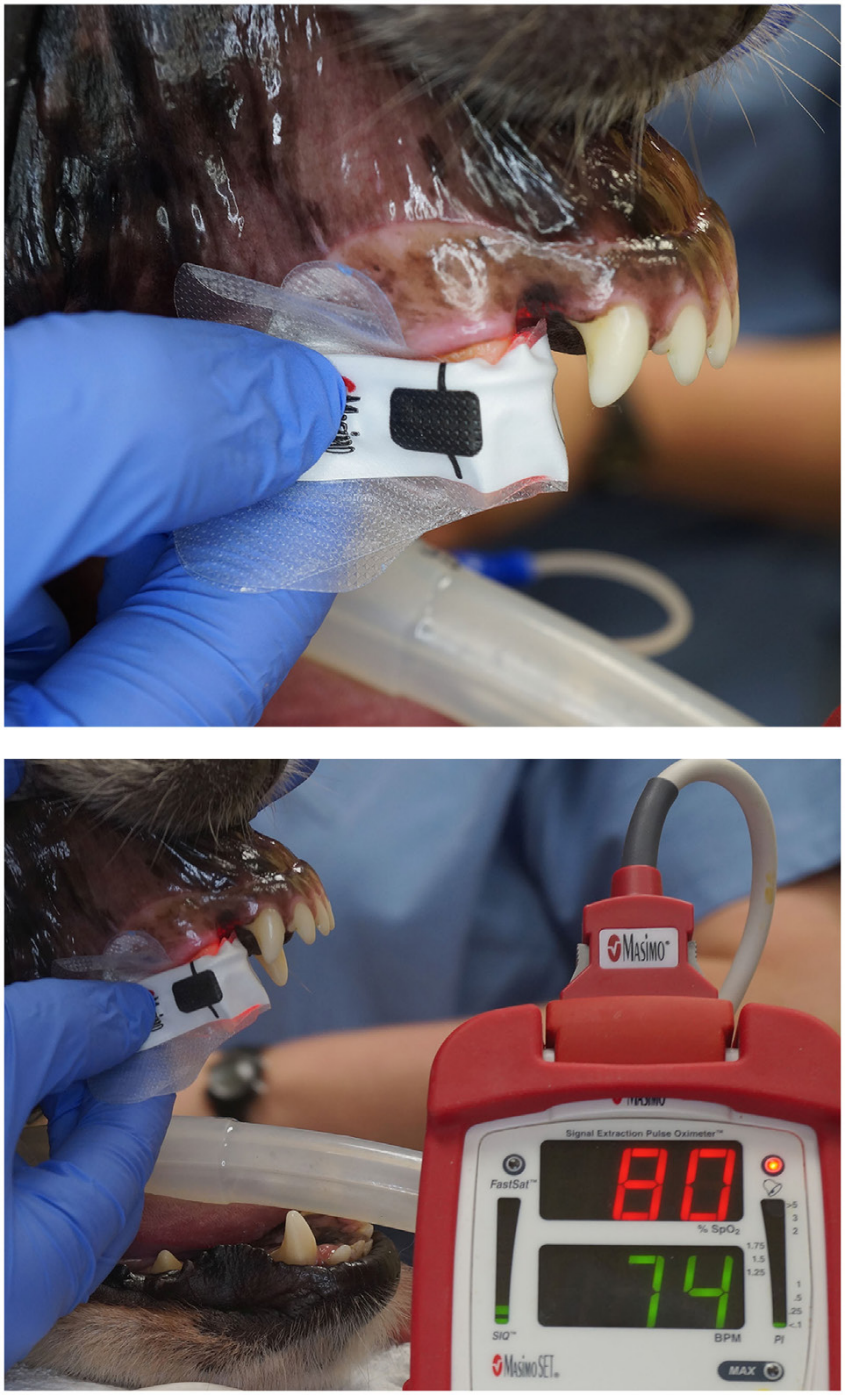
**Photographs depicting the adhesive finger sensor placed on a vital canine tooth**. Note that the opaque shield used to prevent contamination from ambient light has been removed for illustration purposes.

**Figure 5:**
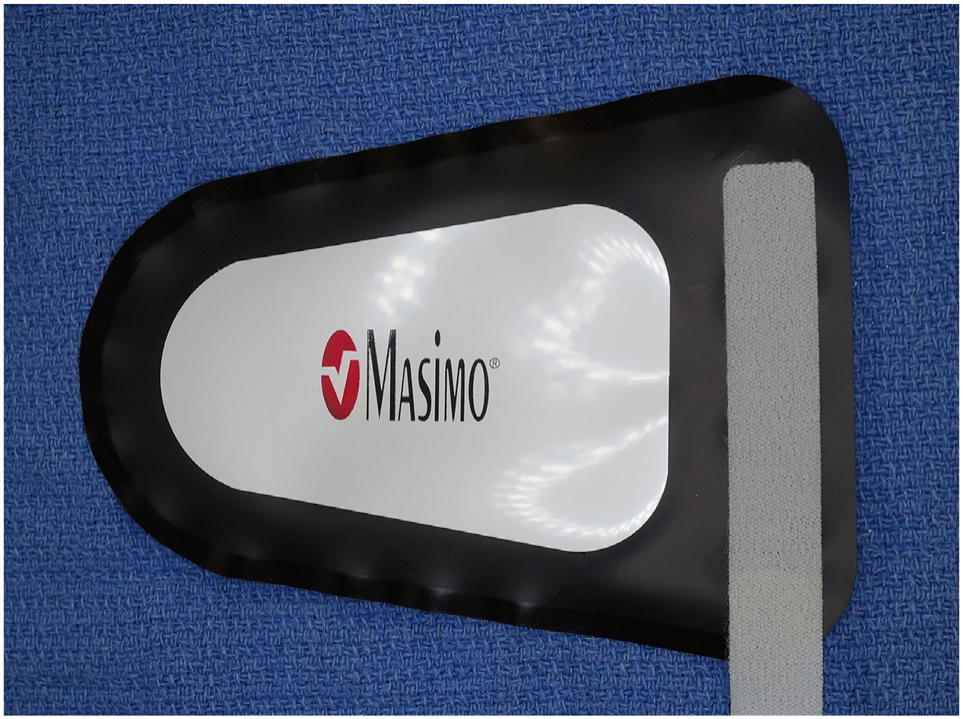
**Photograph of the opaque accessory shield utilized in the study to block penetration of ambient light**.

The study was performed on 40 teeth from 38 anesthetized patients, aged 18–138 months. The anesthesia protocol for each patient was chosen based on the anesthesiologist’s preference. All dogs received anesthetic monitoring and support, consistent with the medical standard and protocols of the hospital. Dogs were divided into three groups by age: juvenile/young adult (11–48 months) (*n* = 12), adult (49–96 months) (*n* = 15), and mature (97 months and greater) (*n* = 13). The study for each dog began after completion of a complete oral examination, dental cleaning, and provision of any requisite oral treatment. This study was carried out using a Rad-5 handheld pulse oximeter^1^ combined with three different sensors. A reusable clip-tip ear sensor^2^ was used to measure the SpO_2_ on the dog’s tongue (Figures 1 and 2). A reusable adult transflectance forehead sensor^3^ was used to measure the SpO_2_ on the dog’s gingiva (Figures 1 and 3). Finally, an adult adhesive finger sensor^4^ was used to measure SpO_2_ on the teeth (Figures 1 and 4). All sensors were calibrated prior to use in the study. The sensor was placed on the teeth (Figure 4) and shielded from ambient light with a manufacturer-provided opaque accessory shield^5^ (Figure 5) to prevent signal interference. Corresponding segments of the sensor were positioned on the labial surface and on the palatal (lingual) surface of the tooth, maintaining parallel alignment between the two diodes, such that the light moved from the labial to the palatal surface of the tooth. Subsequently, SpO_2_ values from the tooth and the control areas (tongue ± gingiva) were recorded every 5 s for three 60-s intervals. The sensors were repositioned after each 60-s interval. All dogs had recordings from teeth and tongue, 22 of which had recordings performed on the gingiva.

### Statistical Methods

Data were summarized using means (±SD) and frequencies of data acquisition (%) for each location separately. Comparisons of SpO_2_ and PR between vital tooth and control (gingiva or tongue) utilized repeated measures ANOVA (RM-ANOVA) to compare the mean values while accounting for within-subject variability. Chi-square tests were used to compare the rate of successful readings of SpO_2_ and PR between vital tooth and control area. For comparisons, based on age group and measurement area, an RM-ANOVA model was used with age group, measurement area, and their interaction as fixed effects, subject as a random effect, and either SpO_2_ or PR as outcome variables. A mixed-effects logistic regression (MELR) model with age group as a fixed effect, subject as a random effect, and whether the measurement was obtained or not as the outcome was used to compare measurement detection rate between age groups at the vital tooth area. All tests were conducted at the 0.05 significance level, and *post hoc* pairwise tests were adjusted using Tukey’s honest significant difference (HSD) method.

## Results

Oxygen saturation was detected on 1,429 (99.2%) out of the 1,440 possible measurements, and PR was detected on 1,392 (96.7%) measurements for the control area. In contrast, SpO_2_ was detected in 340 (23.6%) out of the 1,440 possible measurements, and PR was detected in 317 (22.0%) measurements for the tooth. This was a significant reduction in the frequency of measurement detection for both SpO_2_ and PR between the tooth and the control (*p* < 0.001). Using detected values, the estimated between-subject variability for SpO_2_ was 4.2 in the tooth and was much smaller in the control at only 1.6. Similarly, the estimated within-subject variability for SpO_2_ was much larger in the tooth compared to the control at 7.0 and 0.9, respectively.

There was a significant difference (Wilcoxan signed-rank test, *p* < 0.001) between the time required to acquire a reading from the tooth and the control area. The median [interquartile range (IQR)] time to acquire the first reading from each 60-s testing interval for the tooth was 5 s (5–10 s). The median (IQR) time to acquire a reading in the control area was 5 s (5–5 s).

**Figure 6:**
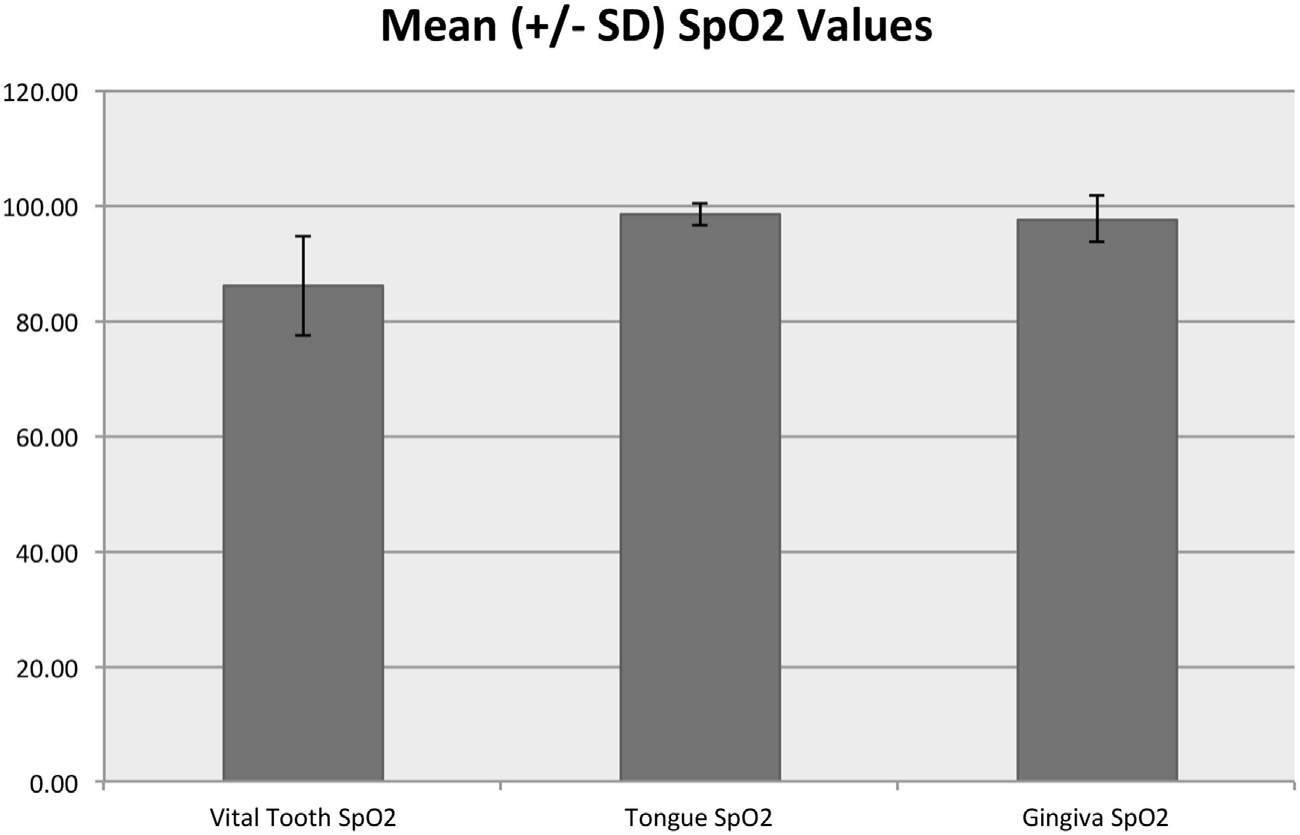
**Mean (±SD) SpO_2_ values for vital teeth and control areas**. The *y*-axis values are reported as percentage.

**Figure 7:**
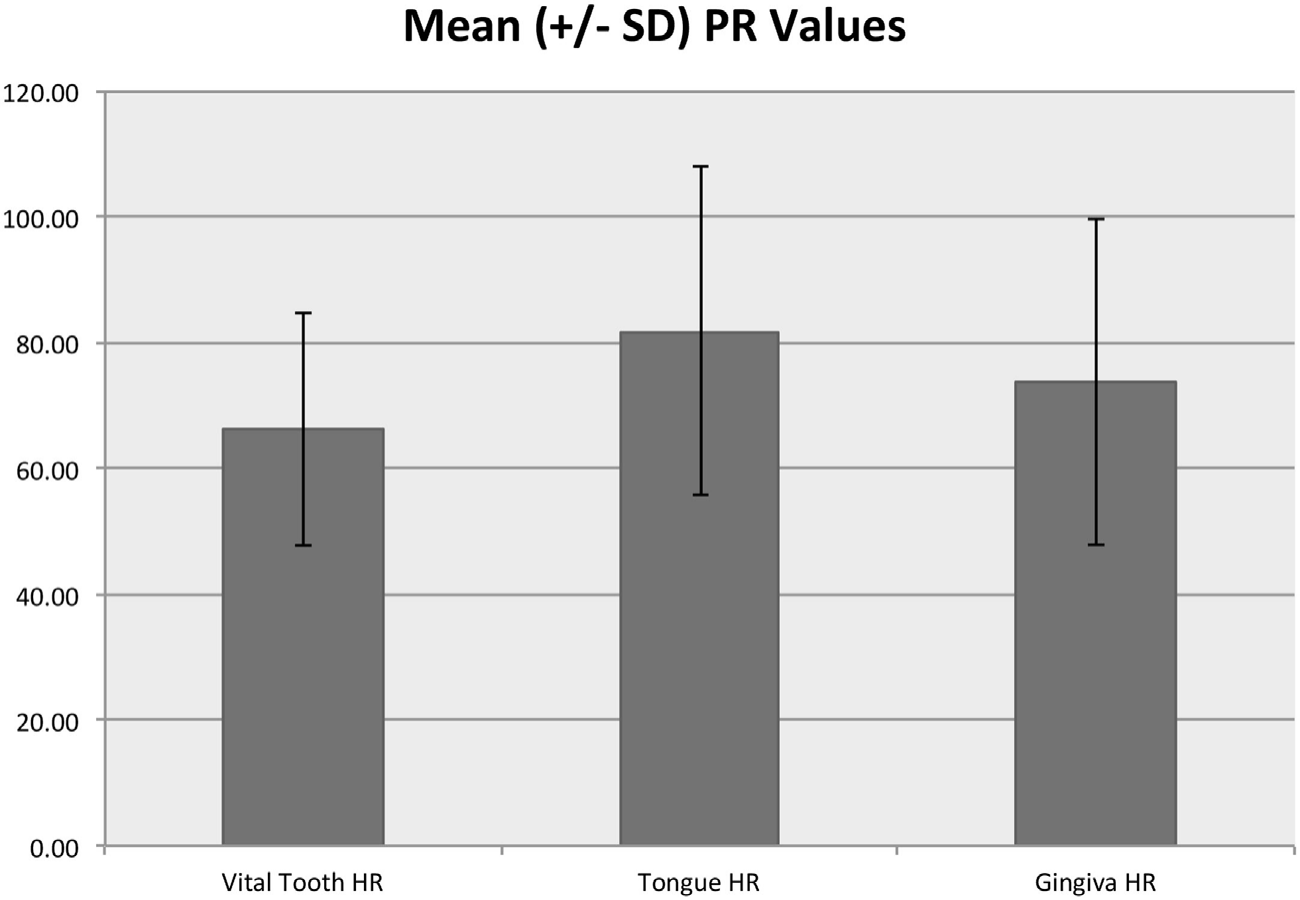
**Mean (±SD) PR values for vital teeth and control areas**. The *y*-axis values are reported as beats per minute.

In the control area, SpO_2_ ranged from 90 to 100% with a median of 98% and mean (SD) of 97.7% (1.8%), while the tooth SpO_2_ was significantly lower (*p* < 0.001) and ranged from 35 to 100% with a median of 87% and mean of 85.9% (8.6%) (Figure 6). Pulse rate acquired from the control area ranged from 25 to 194 beats per minute (bpm) with a median of 71 and mean of 74.7 (26.0) bpm. Pulse rate was significantly lower in the vital tooth (*p* < 0.001) and ranged from 24 to 129 bpm with a median of 65 and mean of 66.3 (18.6) bpm (Figure 7).

**Figure 8:**
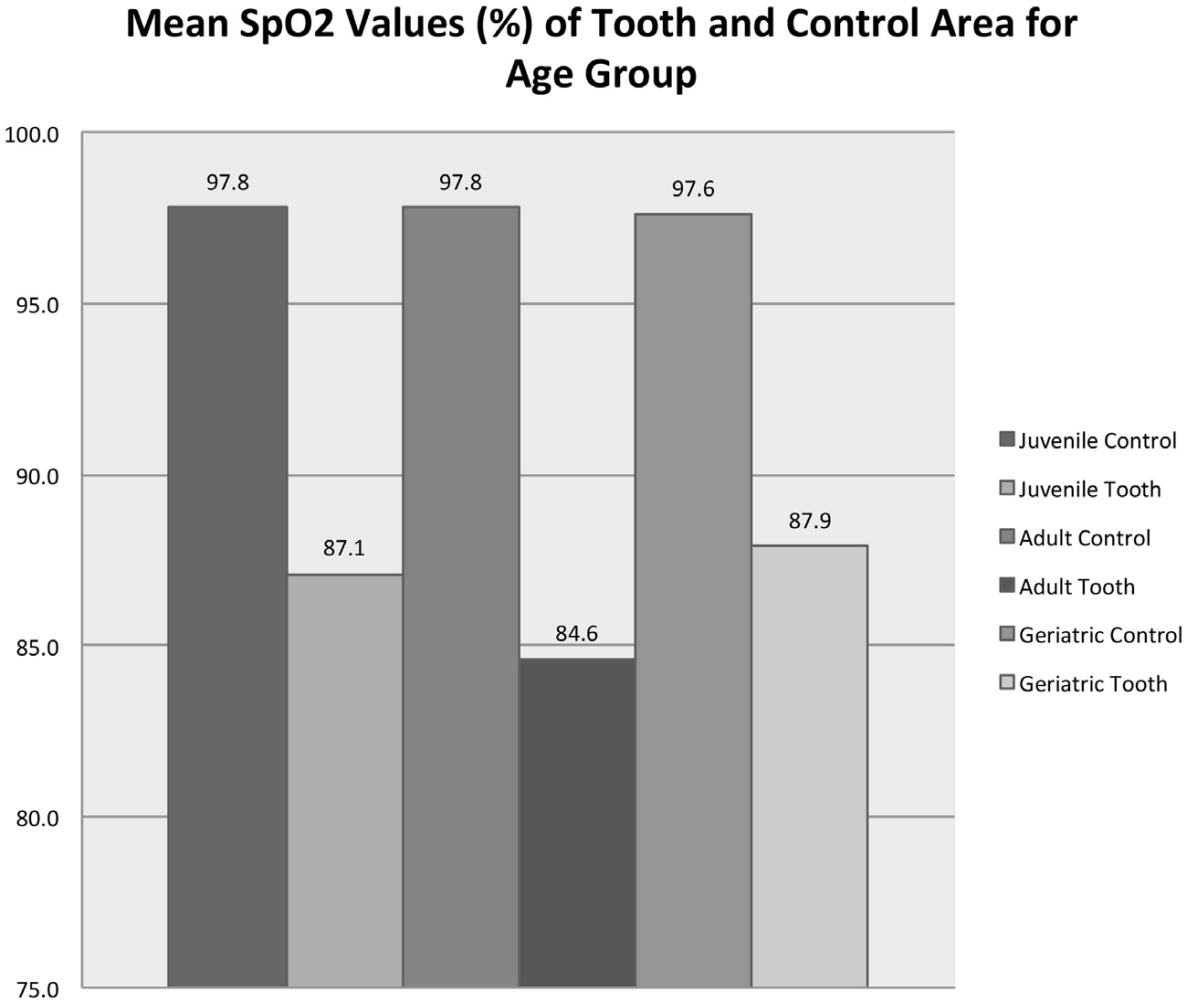
**Mean SpO_2_ values for vital teeth and control areas for each age group**. Note that the control areas (tongue and gingiva) are reported as a singular group for statistical purposes.

**Figure 9:**
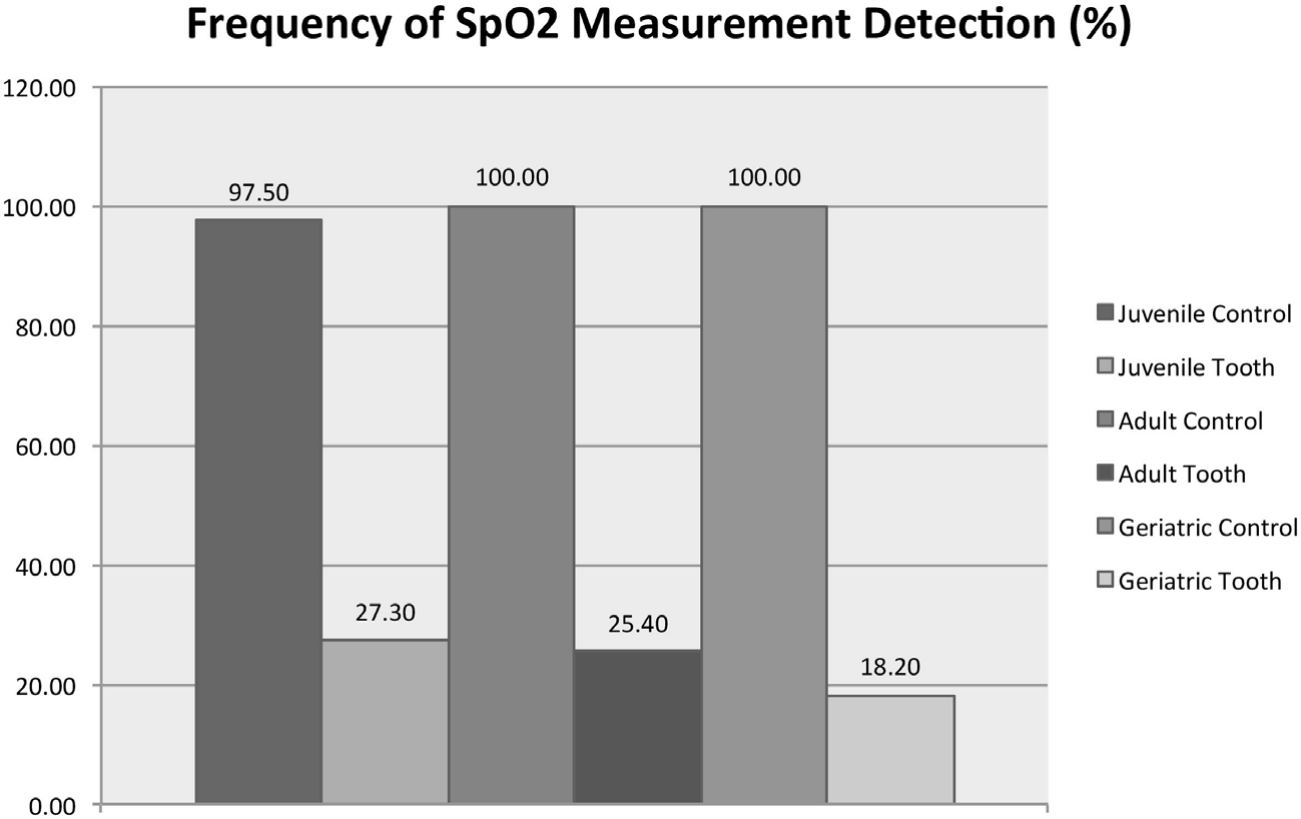
**Detection frequency of SpO_2_ for vital teeth and control areas for each age group**. Note that the control areas (tongue and gingiva) are reported as a singular group for statistical purposes.

Figure 8 shows that age group (juvenile, adult, or mature) significantly affected the average SpO_2_ from the vital tooth (RM-ANOVA, *p* < 0.001) but did not affect the SpO_2_ from the control area (RM-ANOVA, *p* = 0.712). All control/age group combinations are significantly different than all vital tooth/age group combinations (all Tukey HSD, *p* < 0.001). Vital tooth SpO_2_ was significantly lower in adult dogs compared to juvenile (Tukey HSD, *p* = 0.030) and mature dogs (*p* = 0.002), while there was no difference between juvenile and mature dogs (Tukey HSD, *p* = 0.921). Figure 9 shows that age group did not significantly affect the frequency of SpO_2_ detection from the vital tooth (MELR, *p* = 0.430) and also it did not affect the frequency of SpO_2_ detection from the control area.

Table 1 shows that age group significantly affected the average PR measurements (RM-ANOVA, *p* < 0.001). After Tukey’s HSD *p*-value adjustments, the only statistically significant differences in PR are between control and vital tooth locations for adult dogs and mature dogs (both Tukey’s HSD, *p* < 0.001). There were marginal differences in PR between adult dogs from the tooth compared to mature dogs from the control area (Tukey HSD, *p* = 0.059). Age group did not significantly affect the frequency of PR detection from the vital tooth (MELR, *p* = 0.480) and also it did not affect the frequency of PR detection from the control area.

**Table 1:** **Mean PR from vital teeth and control area according to age group**.

Area	Age group	PR (bpm)^a^
Control	Juvenile	75.6 (61.6–89.5)
Control	Adult	66.0 (53.0–78.9)
Control	Mature	83.4 (70.0–96.9)
Vital tooth	Juvenile	78.3 (64.2–92.4)
Vital tooth	Adult	56.2 (43.2–69.3)
Vital tooth	Mature	75.4 (61.8–89.0)

*^a^Reported as estimated mean (and 95% CI)*.

## Discussion

The results of the present study found a consistent yet significantly lower mean SpO_2_ and mean PR for vital teeth when compared to the control area. The difference between the mean values for vital teeth and mean values for the control area represented a 12.1 and 11.24% reduction in SpO_2_ and PR values, respectively. The median SpO_2_ value acquired from teeth in the present study was 87% (mean = 85.9%; SD = 8.6). These findings are consistent with other studies in humans (20, 22, 26, 30). In a study of maxillary central incisors, Calil et al. showed similar consistency between mean SpO_2_ values obtained from the index finger and the vital teeth, ranging between 90.6 and 91.4%, dependent upon the tooth of interest (26). The mean SpO_2_ value in the control area was 95%. The difference in values between the index finger and tooth was 4.2%, with the vital tooth values consistently lower than the index finger. Earlier human studies found similar consistency and only slightly lower SpO_2_ values in vital teeth when compared to control areas. Schnettler and Wallace reported a mean SpO_2_ value of 94% in the maxillary central incisor representing a 3.1% drop in values when compared to a finger (22). In another study of primary and immature permanent maxillary central and lateral incisors, Goho reported a 4.12 and 3.41% lower SpO_2_ reading for primary and immature permanent incisors, respectively, when compared to the index finger (17). The mean values for vital teeth were 94% (SD = 2.25) and 93% (SD = 3.23) for immature permanent and primary incisors, respectively (17).

The SpO_2_ values acquired from vital teeth in the present study (median = 87%; mean = 85.9%; SD = 8.6) are similar to the results from several more recent human studies. In a study of permanent maxillary central incisors, Gopikrishna et al. reported a SpO_2_ range of 75–85% (20). A range of 80–93% (mean 86.7%; SD = 3.26) was reported by Karayilmaz and Kirzioglu in a study of maxillary central and lateral incisors (30). Furthermore, Munshi et al. found that mean SpO_2_ readings for vital right and left maxillary central incisors and right and left maxillary lateral incisors were 81% (SD = 1.68), 81% (SD = 1.34), 80.52% (SD = 1.31), and 80.75% (SD = 1.99), respectively (25). Pozzobon et al. reported similar values with significant differences between vital teeth and the index finger (28). As evidenced by the SpO_2_ range and SD reported in the present study and in previous human studies, variability in SpO_2_ values acquired from teeth should be higher than those values acquired from the control area. The present study also found that this variability exists both within subjects (SD = 7.0) and between subjects (SD = 4.2).

Our results also indicate that the acquisition of a reading from the tooth can be significantly delayed. Data acquisition from the control area consistently occurred within 5 s. However, acquisition from the tooth generally took twice as long when detected and occasionally took up to 60 s. This is consistent with one human study in which the mean acquisition times for primary central incisors and primary canine teeth were 58.04 s (SD = 18.01) and 64.07 s (SD = 20.34), respectively (28). Although, the mean acquisition times for permanent teeth was not reported in the study by Pozzobon et al., it was noted that the acquisition time was significantly higher compared to primary teeth (28).

Additionally, in the present study, readings for SpO_2_ and PR were detected at a significantly lower frequency in teeth when compared to the control area. We recorded readings from the control area and the tooth every 5 s for three 60-s intervals. Most previous studies simply utilized a single recording as proof of test applicability rather than attempt to acquire multiple readings as was attempted in the present study. Therefore, it is difficult to interpret what a low detection frequency actually represents. We cannot say definitively whether this finding substantiates either tooth vitality or tooth necrosis. The summary of data collected in the present study, which is consistent with data from the human studies, tends to support the concept that a single reading, acquired within a 60- to 90-s period that can also be repeated, may be a reliable indicator of tooth vitality.

In general, SpO_2_ readings from teeth were quantitatively lower, were detected with lower frequency, had a longer acquisition time, and proved more variable than readings acquired from the control area. Several potential explanations for these findings can be developed by first understanding the functionality of pulse oximeters. The pulse oximeter was first developed by a Japanese bioengineer, Takuo Aoyagi, in 1974 and is based on the Beer–Lambert law, which states that an unknown concentration of solute (hemoglobin) dissolved in a known solvent (blood) can be assessed by the light absorption of the solute (27, 31–34). Accordingly, there is dependence between light transmitted through a substance and the product of the absorption coefficient of the substance and the distance light travels through the material that is logarithmic in nature. The pulse oximeter sensor contains a light-emitting diode (LED) that transmits red light (~660 nm) and an LED that transmits infrared light (900–940 nm). Variable absorption of the red and infrared light occurs by oxygenated and deoxygenated hemoglobin. The remaining light passes through the tissue of interest and is received by a photodetector diode. Pulsatile changes in blood volume causes alterations in the amount of red and infrared light absorbed by the vascular tissue prior to reaching the photodetector diode. The relationship between the pulsatile changes in the absorption of the light is processed by a microchip in the pulse oximeter to display a value for SpO_2_.

In order for pulse oximeter sensors to optimally perform, the sensor should conform to the contours of the tissue being evaluated. Additionally, the LED and photodetector on either side of the tooth should be maintained as parallel as possible to one another in order to ensure proper functioning of the sensor. In the present study, we used a sensor that had flexible LED and photodetector sides in order to ensure that the leads conformed to the contours of the tooth. However, in order to avoid any penetration of ambient light, we also covered the sensor with an opaque plastic shield. This step may have introduced error in that we could no longer visualize the sensor to ensure that the LED and photodetector followed the contours of the tooth or were adequately parallel to one another and the tooth. As other researchers have noted, the development and manufacture of specific sensors for dental use would help alleviate any concern of poor sensor conformation or parallelism. Some researchers have utilized custom-made sensors or jigs to hold commercially available sensors (20, 22, 25, 28), and we would consider their application in future studies.

Teeth present a unique challenge to the successful use of diagnostic modalities that utilize a light source. It has been proposed that enamel prisms and dentin may cause diffraction of the infrared light (25, 35) or may scatter light rays through the gingiva (36). The poor correlation between tooth SpO_2_ and control area SpO_2_ seen in the present study and reported in other studies could be attributed to this phenomenon. Calil et al. reported that, in order to capture a more reliable pulse signal in the tooth, amplification of the pulsing signal through modification of the amplifying circuits of the pulsing element of the equipment was necessary (26).

Pulse oximetry accuracy also requires normal peripheral arterial blood flow. When arterial pulsatile blood flow is low, as may occur with hypovolemia, hypothermia, and significant peripheral vasoconstriction, pulse oximetry readings may be artificially altered or may become unobtainable. While the anesthetic plan for all patients in the present study was managed by a board-certified anesthesiologist and all patients maintained at clinically acceptable blood pressure throughout the testing, it is possible that some patients may have experienced momentary peripheral vasoconstriction during tooth SpO_2_ reading acquisition, which may have influenced the ability to acquire a reading. Additionally, the lack of a standard anesthetic regimen between patients and the inability to correlate pulse oximetry readings with blood pressure readings may have contributed to a reduction in reading detection.

In the present study, tooth SpO_2_ values were significantly lower in adult dogs compared to juvenile and mature dogs. However, there were no significant differences in tooth SpO_2_ values between juvenile dogs and mature dogs. Additionally, the age of patient and the ability to detect SpO_2_ readings were not correlated. This is contrary to what we would expect. If dentin thickness decreases the likelihood of light penetrations (36), we would expect detection ability and quantitative values to decrease with age. While we did see values that were lower in adult dogs compared to juvenile dogs, we would not expect mature dog values to be higher than adult dog values.

The authors would like to acknowledge some limitations with the present study. Vitality of the teeth included in the study was confirmed based on the lack of radiographic evidence of pulp necrosis. As noted in the Section “Introduction” of this manuscript, radiologic evidence of pulpal necrosis is an end-stage feature. Thus, it is possible that some non-vital teeth were included in the study, which would introduce error into the results. Lately, computed tomography (CT) has been shown to be more effective than traditional radiography at detecting subtle periapical lucencies (37). Thus, future studies that intend to screen for pulpal health may consider the incorporation of CT. However, the only way to absolutely confirm pulp vitality would be to perform histopathology of the teeth, which was considered impractical and unethical. Additionally, as noted above, the anesthetic regimen was not standardized between patients. However, given the subjects were client-owned animals with various anesthetic requirements, this was considered impractical. In retrospect, it may prove useful to coordinate the SpO_2_ acquisition from the tooth with recordings of systemic blood pressure acquired with arterial catheterization to ensure appropriate peripheral arterial blood flow. Finally, although we attempted to assess the influence of dentin thickness on oxygen saturation of the pulp by including patients of increasing age, in retrospect, perhaps assessing the influence of tooth size may also have been evaluated.

This study presents findings that support the potential use of pulse oximetry as a diagnostic tool for determining pulp vitality. We demonstrated that the described method determined the SpO_2_ level of the vital canine tooth pulp. No statistical correlation was found between the SpO_2_ level obtained from the control area and that obtained from the teeth. However, SpO_2_ values acquired from the teeth, when detected, were found to be consistent with previous reports in humans. Although the findings in the study reported here support the possible use of pulse oximetry to detect pulpal blood flow in dog canine teeth, no definitive conclusion on the overall clinical usefulness of pulse oximetry can be provided as of now, yet future studies to quantify SpO_2_ values in non-vital teeth are necessary. Further support for the use of pulse oximetry to evaluate pulpal blood flow in dog canine teeth could be given, if future studies observe non-vital teeth to have consistently lower SpO_2_ values that are significantly different than the values presented here for vital teeth.

## Author Contributions

All authors listed have contributed sufficiently to the project to be included as authors, and all those who are qualified to be authors are listed in the author byline.

## Conflict of Interest Statement

The authors declare that the research was conducted in the absence of any commercial or financial relationships that could be construed as a potential conflict of interest.

## Footnotes

^1^Rad-5 handheld pulse oximeter, Masimo Corporation, Irvine CA, USA.

^2^LNCS TC-I clip-tip ear sensor, Masimo Corporation, Irvine, CA, USA.

^3^LNCS TF-I adult transflectance forehead sensor, Masimo Corporation, Irvine, CA, USA.

^4^M-LNCS Adtx adult adhesive finger sensor, Masimo Corporation, Irvine, CA, USA.

^5^Disposable ambient light shield, Masimo Corporation, Irvine, CA, USA.
